# Extracerebral choroid plexus papilloma in the pharynx with airway obstruction in a newborn: a case report

**DOI:** 10.1186/s12887-020-02234-2

**Published:** 2020-07-07

**Authors:** Narae Lee, Mi Hye Bae, Young Mi Han, Kyung Hee Park, Jae-Yeon Hwang, Cheong-Soo Hwang, Jin-Choon Lee, Shin Yun Byun

**Affiliations:** 1 Department of Pediatrics, Pusan National University School of Medicine, 20 Geumo-ro, Yangsan, 50612 Republic of Korea; 2 Department of Radiology, Pusan National University School of Medicine, Yangsan, Republic of Korea; 3 Department of Pathology, Pusan National University School of Medicine, Yangsan, Republic of Korea; 4 Department of Otorhinolaryngology and Head-neck surgery, Pusan National University School of Medicine, Yangsan, Republic of Korea

**Keywords:** Choroid plexus papilloma, Airway obstruction, Newborn, Case report

## Abstract

**Background:**

Choroid plexus papillomas (CPPs) are rare, usually benign, neoplasms originating in the central nervous system. In this study, we present the first case of a giant airway-obstructing CPP in the pharynx of a newborn.

**Case presentation:**

A cystic mass located in the pharynx was noted in a fetus at the 29th week of gestation. Elective cesarean section was performed at the 38th week of gestation with successful intubation and ex utero intrapartum treatment. On computed tomography, there was a huge airway-obstructing cystic mass in the choana and pharynx. Elective surgery with total excision was performed, and histological examination confirmed the diagnosis of CPP.

**Conclusion:**

We report the first case of an extracerebral airway-obstructing CPP in the pharynx of a newborn. Radiologic examinations are not enough for the diagnosis of CPPs, and complete excision of the tumor with histological confirmation is indispensable for accurate diagnosis and treatment.

## Background

Choroid plexus papillomas (CPPs) are rare tumors originating from the central nervous system. Almost all CPPs are histologically benign neoplasms. Its prevalence rates in adult and children are 0.5–1% and 3–4% of primary intracranial neoplasms, respectively [1, 2]. The papillae consist of cores of fibrovascular tissue; the choroid plexus is a neuroepithelial-lined papillary projection of the ventricle ependymal [3]. Therefore, most CPPs occur in the ventricular system. In the majority of cases, an increased intracranial pressure is the most common presentation, and other symptoms are dependent upon the site of lesion [4]. The cerebellopontine (CP) angle, third ventricle, and cerebral parenchyma are rare locations [1]. In this report, we present the first case of a giant extracerebral CPP in the pharynx with airway obstruction in a newborn.

## Case presentation

A 30-year-old woman undergoing regular follow-ups at an antenatal clinic and no treatment with any medicines except iron supplementation presented to our clinic. In the 29th week of gestation, a cystic mass approximately 1.8 × 2.0 cm in size located in the pharynx was noted on a routine ultrasound examination of the fetus. One month later, follow-up sonography revealed the lesion to have the same size. The amniotic fluid index (AFI) was 21.43 cm in the 30th week of gestation and increased up to 24.2 cm in the 34th week of gestation, and polyhydramnios was defined as AFI > 24 cm. There was a possibility of intubation failure. We planned an elective cesarean section with ex utero intrapartum treatment (EXIT) for prolonged stabilization of the fetal hemodynamic environment at the 38th week of gestation. A multidisciplinary team was assembled involving 2 neonatologists, 2 otorhinolaryngologists, 2 obstetricians, and 4 scrub nurses. A neonatologist performed successful intubation immediately after exposure of the fetal body, followed by umbilical cord clamping and delivery of the baby. The patient presented at birth with stable vital signs during endotracheal intubation.

The female neonate was the first child born to the 33-year-old father and 30-year-old mother. The Apgar scores at 1 and 5 min were 7 and 8, respectively. She had a body weight of 2330 g (3rd percentile), height of 45 cm (25th percentile), and a head circumference of 32 cm (20th percentile); thus, she was diagnosed with asymmetric intrauterine growth restriction (IUGR). On the third day of life, the TORCH screening was done with serum immunoglobulin M (IgM) of t*oxoplasma* and rubella, whole blood polymerase chain reaction (PCR) of Herpes simplex virus-1 (HSV-1) and HSV-2, and real-time PCR of cytomegalovirus from urine, and all were negative.

**Fig. 1:**
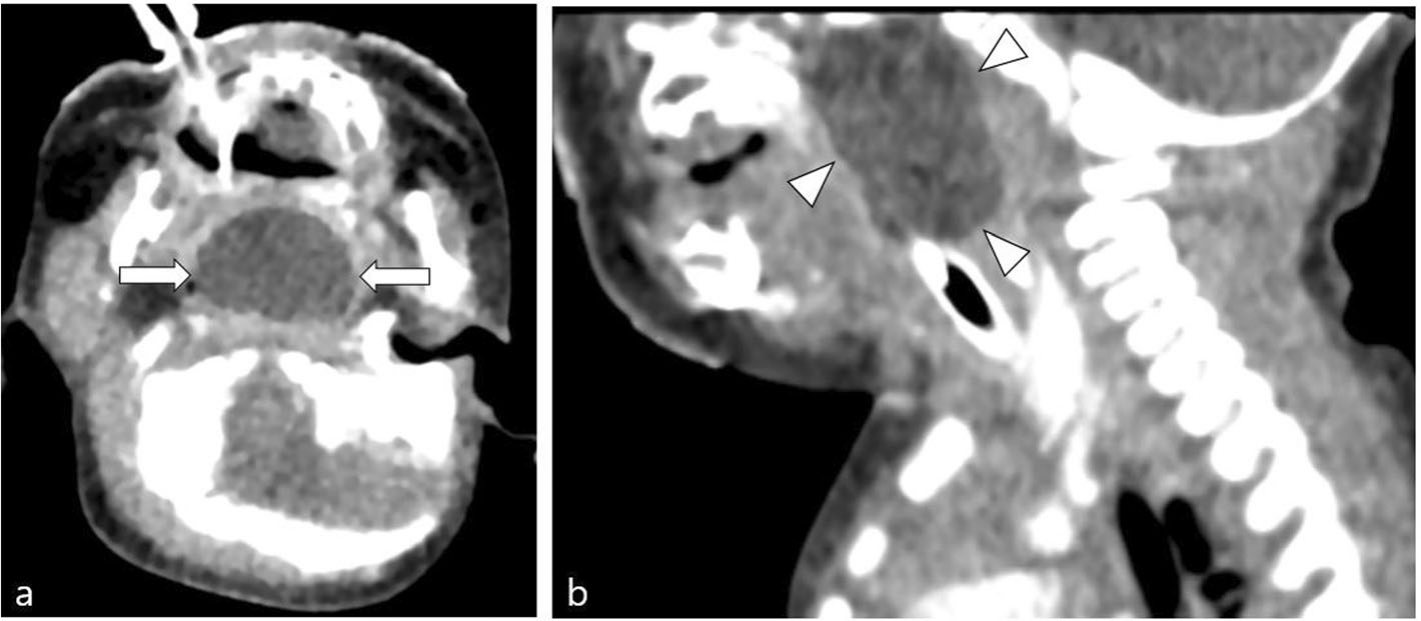
Contrast-enhanced computed tomography (CT) scan of the airway. **a** Axial CT scan obtained at the level of the oropharynx demonstrates a large cystic lesion (2 × 2.8 cm) causing airway obstruction (arrows). **b** Sagittal reconstructed image shows a midline cystic lesion involving the choana, nasopharynx, and oropharynx (arrowheads)

**Fig. 2:**
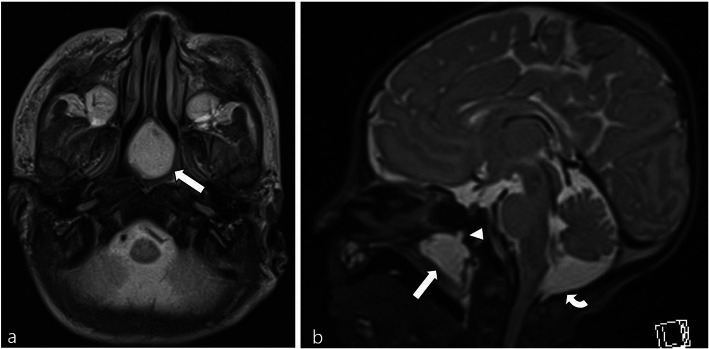
Precontrast magnetic resonance imaging of the brain. **a** An axial T2-weighted image and **b** a sagittal T2-weighed image showing a cystic mass at the choana and nasopharynx causing airway obstruction (arrows). Note a 4-mm fistula-like structure in the sphenoid bone (arrowhead). There was no discernible communication with intracranial structures. Further, a mega cisterna magna was incidentally detected (curved arrow)

A 3D computed tomography (CT) of the chest airway performed shortly after birth showed airway obstruction owing to a cystic mass in the choana, nasopharynx, and oropharynx (Fig. 1); we could not exclude the diagnosis of an encephalocele. Non-contrast brain magnetic resonance imaging (MRI) was performed for further evaluation at the age of 10 days old; MR protocol included axial T2-weighted image, axial T2 fluid attenuated inversion recovery (FLAIR), axial diffusion-weighted image, susceptibility-weighted image, and three-dimensional T1- and T2-weight image with multiplanar reconstruction. On the MRI, the mass showed a 4-mm fistula-like structure directed to the sphenoid bone; however, there was no definite communication with intracranial structure (Fig. 2).

**Fig. 3:**
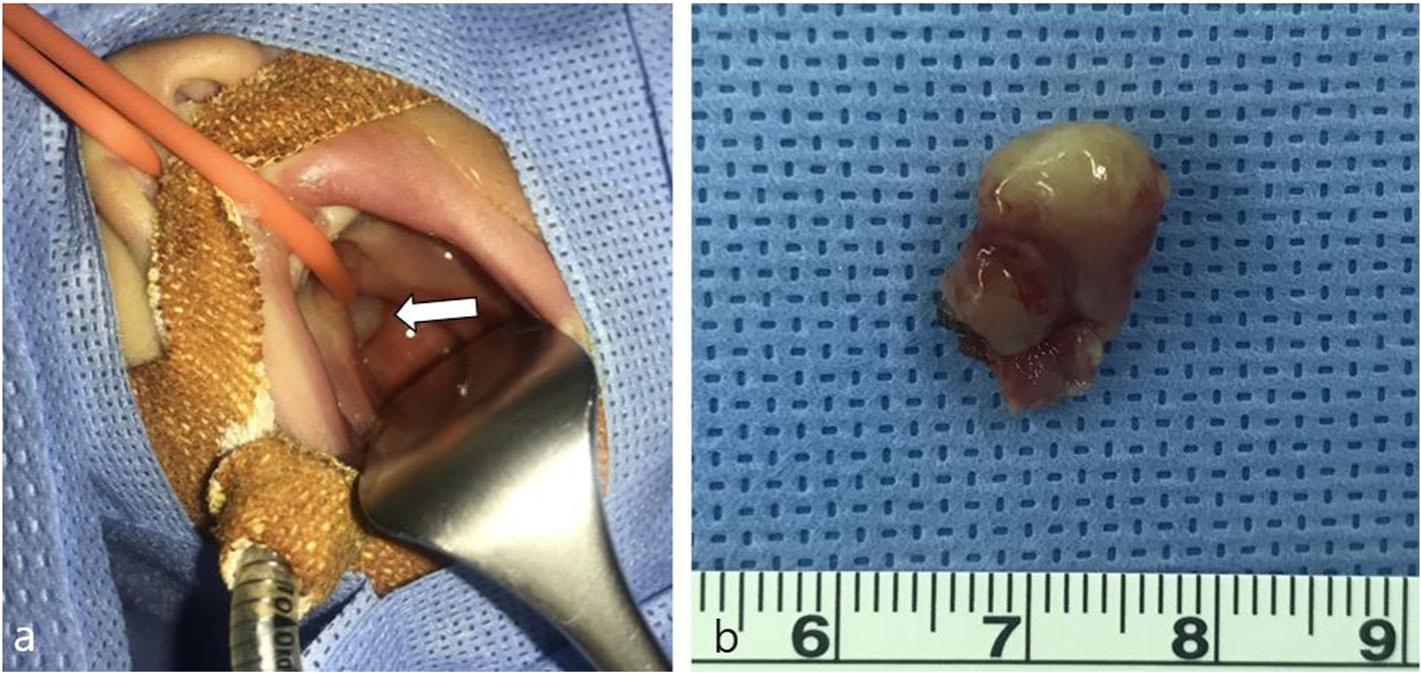
**a** Whitish protruding mass between the prevertebral fascia and nasal septum in the transoral view (arrow). **b** Gross tissue

**Fig. 4:**
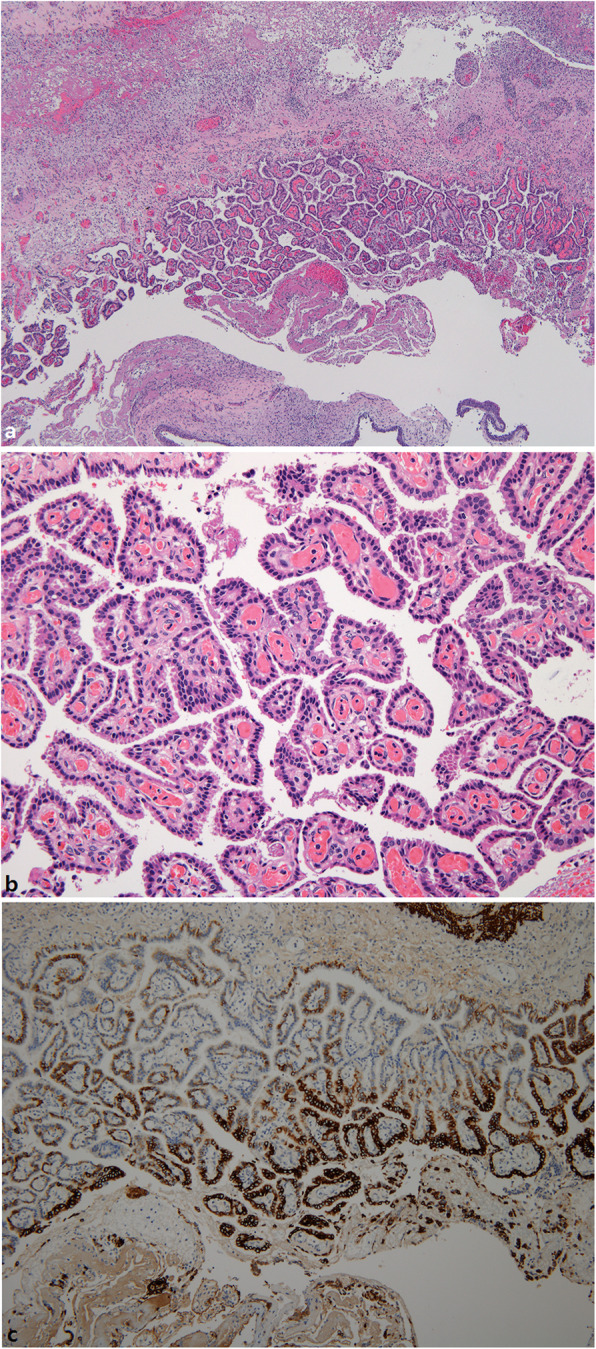
**a** A cystic mass was lined by complex papillae of epithelial cells and walled with glial and fibrovascular tissues (H&E, 40×). **b** The complex papillae of the cyst comprised a single layer of epithelial cells resembling epithelial cells of the choroid plexus tissue and fibrovascular cores (H&E, 200×). **c** The choroid plexus-like cells were focally positive for GFAP immunostain (Immunoperoxidase staining, 100×)

An elective surgery was performed at 16 days of age by otorhinolaryngologists. Under general anesthesia, removal of the pharyngeal mass through a transoral approach was performed in the Rose position. A whitish, protruding mass adhered to the prevertebral fascia and nasal septum, but was easily dissected to complete extirpation. The mass was 1.6 × 1.1 × 0.4 cm in size and 0.3 g in weight (Fig. 3). Histological examination at low magnification revealed that the mass was a cystic lesion with complex papillary proliferation of epithelial cells. The wall of the cyst was composed of glial and fibrovascular tissues (Fig. 4a). At high magnification, the papillary components of the cyst comprised a single layer of epithelial cells and fibrovascular cores. Epithelial cells were cuboidal-to-columnar in shape and had round-to-oval and basally located nuclei with an eosinophilic cytoplasm, resembling epithelial cells of the choroid plexus tissue. Cytologic atypia or mitotic activity was not identified (Fig. 4b). The histologic features of the tumor were consistent with those of a CPP, arising from glial heterotopia. The choroid plexus-like cells showed focal positivity for Glial fibrillary acidic protein (GFAP) immunostain (Fig. 4c). Pathology analysis was consistent with WHO grade I CPP. Extubation was performed at 2 days after surgery, and the respiration rate and peripheral saturation were stable. Seven months post-surgery, a non-enhanced CT of the pharynx was performed and resection site was clear. Chromosome analysis revealed a normal female karyotype with 46, XX at 7 months of age. Currently, the patient is doing well without any symptoms until 10 months.

## Discussion and conclusions

CPPs were first reported in 1832 by Guerard in a 3-year-old child [5]. In 2016, the World Health Organization (WHO) classified choroid plexus tumors as CPP (WHO Grade I), atypical CPP (WHO Grade II), and choroid plexus carcinoma (WHO Grade III) [6]. CPPs are well-known benign neoplasms of the central nervous system, accounting for approximately 0.4–0.6% of all intracranial tumors [7]. Stemming from the neuroectoderm and originating from the choroid plexus lining the ventricles, overproduction of cerebrospinal fluid (CSF) is a critical feature of these tumors [1, 2]. Most of them are located in the atrium of the lateral ventricles in children, with some cases being reported in the fourth ventricle [8, 9]. The posterior third ventricle and CP angle are rare locations [1, 10, 11].

To our knowledge, we report the first case of an extracerebral CPP in the pharynx presenting with airway obstruction that was resected completely without complications. Newborns are usually considered obligate nasal breathers despite being anatomically capable of breathing orally. For this reason, congenital nasal masses can cause respiratory distress in newborns. In infancy, the most common midline nasal masses are dermoids, encephaloceles, and gliomas [12]. Nasal dermoids are a result of a defect in development of the anterior neuropore with ectodermal and mesodermal derivatives and contain epithelium, sebaceous tissue, and/or hair. Encephaloceles and gliomas are derived from defects in the skull base; however, gliomas do not communicate with the subarachnoid space [12]. In this case, immediately after confirmation of the MRI results, our patient was thought to have the typical clinical and radiological features of a dermoid because there were no defects in the skull base and no connection with the subarachnoid space. The mass also spontaneously decreased in size with time. On MR images, most of CPPs appear as isointense to hypointense intraventricular masses, and following contrast injection, they demonstrate notable homogeneous or heterogeneous enhancement, because typical CPPs are known as hypervascular tumors [13]. However, the misdiagnosis rate of extraventricular CPPs is high as other diseases, including meningioma, neurinoma and ependymoma [13]. Limitation of this case is that did not performed enhanced MRI, but in the preoperative contrast-enhanced CT scan showed purely cystic legion without enhancing portion. Therefore, in this case, it is very difficult to diagnose CPP through MRI.

The pathological diagnosis of CPPs is similar to normal nonneoplastic choroid plexus tissue. Fibrovascular core lined by a single layer of columnar or cuboidal cell is typical finding [14]. In case of showing hypercellularity, conspicuous mitotic activity, and invasion into the brain parenchyma can be diagnosed with the choroid plexus carcinoma [14]. The expression of p53 is to be also diagnosed as choroid plexus carcinoma but undetected in the CPPs [15]. Positive of cytokeratin, S-100 and vimentin are well documented in CPP, and the absence of Epithelial Membrane Antigen (EMA) and GFAP further favors the diagnosis of CPP [16]. In this case, there is typical WHO grade I CPP, and no anaplastic features identified. It also does not contain mature astrocytes or gliosis. On immunohistochemistry, EMA was negative, GFAP shows focal positive, and cytokeratin, S-100 and vimentin stains were not inspected.

In the treatment of CPPs, complete surgical resection is recommended and essential for an accurate diagnosis. The survival rates associated with CPP after surgery are high. A meta-analysis revealed that the 1-, 5-, and 10-year survival rates associated with CPP are 90, 81, and 77%, respectively [17]. No adjuvant treatment is required, except in atypical and aggressive cases [1].

Obstetric ultrasonography is important for reducing the risk of unexpected poor outcomes or death of newborns. In this case, prenatal sonographic diagnosis of a slowly growing tumor, which can lead to respiratory distress owing to airway obstruction, allowed for the preparation for an EXIT process. A period of sustained utero-placental gas exchange was required to partly deliver the baby, expose the trachea, and reverse the airway occlusion. This procedure can be used to treat a variety of respiratory-distress conditions at delivery because of the prolonged stable fetal hemodynamic environment it provides [18].

In summary, we herein report the first case of extracerebral CPP in the pharynx of a newborn with respiratory distress due to airway obstruction. Prenatal ultrasound is crucial for preventing poor outcomes in infants, and all pediatric patients with a laryngeal mass should undergo CT and MRI to rule-out a tumor with an intracranial connection; however, this is not enough to reach a definite diagnosis. Complete excision of the mass and histological confirmation are both indispensable for accurate diagnosis and treatment.

## Data Availability

All data generated or analyzed during this study are included in this published article.

## Abbreviations

CCP: Choroid plexus papilloma
CP: Cerebellopontine
CSF: Cerebrospinal fluid
CT: Computed tomography
EMA: Epithelial Membrane Antigen
EXIT: Ex utero intrapartum treatment
GFAP: Glial fibrillary acidic protein
H&E: Hematoxylin and eosin staining
HSV: Herpes simplex virus
IgM: Immunoglobulin M
IUGR: Intrauterine growth restriction
MRI: Magnetic resonance imaging
PCR: polymerase chain reaction
WHO: World Health Organization
